# Corrigendum

**DOI:** 10.5713/ajas.2005.892C

**Published:** 2022-04-22

The following is the updated author list of **Asian-Aust. J. Anim. Sci. 2005. Vol 18, No. 6 : 892–897**.

Chung-boo Kang*, Cheol-ho Kim, and Sang-gil Lee

College of Veterinary Medicine, Gyeongsang National University, Jinju 660-701, Korea.

*Corresponding author

